# *Haematococcus pluvialis* Microalgae Extract Inhibits Proliferation, Invasion, and Induces Apoptosis in Breast Cancer Cells

**DOI:** 10.3389/fnut.2022.882956

**Published:** 2022-05-11

**Authors:** Nouralhuda Alateyah, Salma M. S. Ahmad, Ishita Gupta, Arij Fouzat, Mahmoud Ibrahim Thaher, Probir Das, Ala-Eddin Al Moustafa, Allal Ouhtit

**Affiliations:** ^1^Biological Sciences Program, Department of Biological and Environmental Sciences, College of Arts and Sciences, Qatar University, Doha, Qatar; ^2^College of Medicine, Qatar University, Doha, Qatar; ^3^College of Pharmacy, Qatar University, Doha, Qatar; ^4^Algal Technology Program, Center for Sustainable Development, College of Arts and Sciences, Qatar University, Doha, Qatar; ^5^Biomedical Research Centre, Qatar University, Doha, Qatar

**Keywords:** *Haematococcus pluvialis* microalgae, breast cancer, cell proliferation, apoptosis, invasion

## Abstract

Breast cancer (BC) is the most common malignant cancer in females worldwide. Drug resistance, toxicity, and the failure of current therapies to completely cure BC has challenged conventional medicine. Consequently, complementary alternative medicine has become popular due to its safety and efficacy. *Haematococcus pluvialis* (*H. pulvialis*) is a green microalga living in fresh water, and its crude extract is rich of bioactives, including carotenoids, known to inhibit cancer cell growth. In the present study, we investigated the effects of a methanol crude extract called “T1” of *H. pulvialis* on cell growth and migration/invasion of the BC cell line MDA-MB-231 in comparison to the fibroblast control cells. TI significantly suppressed BC cell growth, inhibited migration and invasion and induced apoptosis. Interestingly, apoptosis was mediated by a significant loss of mutant p53 protein, and increased Bax/Bcl2 ratio. Our findings support our hypothesis that T1 exerts its anti-cancer effects by inhibiting BC invasion and inducing apoptosis mediated, at least, *via* the p53/Bax/Bcl2 pathway. Ongoing experiments aim to identify the molecular mechanisms underpinning T1-inhibited BC cell invasion using pre-designed metastasis gene-based array method.

## Introduction

Breast cancer (BC), a worldwide health issue, is the most common malignant cancer in females worldwide, including the State of Qatar (1, 2). Malignant tumors have the capability to metastasize, involving invasion of cancer cells, the most threatening aspect of cancer (3). In conventional medicine (CM), there is no ultimate cure for cancer, although technology has made a major progress, accompanied by significant discoveries in cancer research that have been eventually applied in designing therapeutic strategies against cancer. CM is still facing serious challenges, including the lack of understanding the specific molecular mechanisms associated with cancer development within various groups of patients, drug resistance, and the failures of clinical trials and current therapies to completely cure the disease. Hence, the field of Complementary Alternative Medicine (CAM) is becoming popular and gaining more attention, particularly in the communities where traditional medicine has been practiced for many years (4, 5). CAM treatment method uses extracts derived from seeds, leaves, fruits, and roots of plants; each of these invariably represents a combination of several bioactive compounds characterized by antioxidant, anti-inflammatory, anti-proliferative, and anti-cancer properties (6, 7).

*Haematococcus pluvialis (H. pulvialis)* is a microalgae considered as the richest source of natural super anti-oxidant known as astaxanthin (ATX) (8). ATX is known for its antioxidant activity that reduces free radicals and oxidative stress (8). *H. pluvialis* extract consist of several biochemical molecules including, proteins, carbohydrates, lipids, and carotenoids. In dried *H. pluvialis* biomass carotenoid can accumulate by 5%, of which ATX makes up 90% (9, 10). Under extreme environmental conditions, high illumination, extreme temperature, salt stress, lack of nutrients and a combination of several other factors can stimulate the production of ATX (11). *H. pluvialis* cultivation or life-cycle comprises of two phases, the green vegetative phase and the red non-motile encysted phase (12). During the life-cycle of *H. pluvialis* cells, the green phase shifts to the red phase with a change in the chemical composition of the cellular content (13). While, in the green phase there is accumulation of 1% of lutein and around 20–25% of fatty acids on dry biomass weight (DBW), in the red phase there is accumulation of 1–5% of ATX and 32–37% of lipids on DBW (8). However, under extreme conditions, the red phase of ATX can reach up to ∼8% of DW (14). Of the carotenoids, ATX and lutein are well-recognized as natural antioxidants (13). Interestingly, carotenoids inhibit the proliferation of several cancer cell lines including breast, prostate, melanoma, lung, and leukemia, resulting in cycle-cycle arrest (15, 16), inhibition of the malignant transformation of cancer cells (17) and the induction of apoptosis (18, 19). Furthermore, carotenoids act as an inhibitor of various signaling pathways mediating cell invasion and metastasis (20). In fact, carotenoids showed to decrease expression of MMP-2, MMP-9, N-cadherin, CD44 receptor, and β-catenin (20). It also inhibited the MAPK, NOTCH signaling, PI3K/AKT/NF-κB, Wnt pathway known to control tumor cell progression (20). These data support the anti-cancer properties of carotenoids in inhibiting cell proliferation, migration, and invasion.

To investigate the potent therapeutic and antitumor properties of the T1 extract from carotenoids of the *H. pulvialis* in human BC and its underlying mechanism, we explored the effect of aqueous extract of T1 extract on cell proliferation, cell migration and invasion in the triple negative BC cell line, MDA-MB-231.

## Materials and Methods

### Collection of Microalgae Samples

**FIGURE 1:**
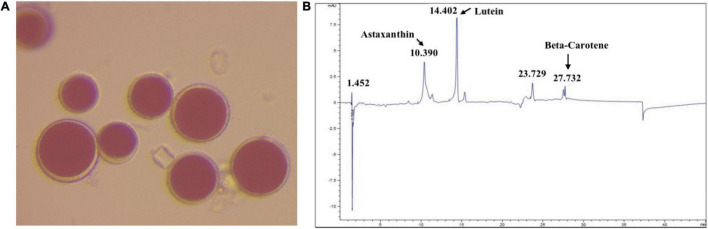
**(A)** Image of magnified *H. pluvialis* culture (magnification, 40×). **(B)** HPLC-chromatograph of the pigments extracted from *H. pluvialis* biomass.

Haematococcus sp., a locally isolated cyanobacterium, was obtained from Qatar University Culture Collection of Microalgae (QUCCM) (Figure 1A). Colonies were transferred to 10 ml liquid medium in 50 ml centrifuge tube. Modified Guillard F2 media supplemented with 3× nitrogen and phosphorus was used. The centrifuge tube was maintained in a growth chamber (PHCbi MLR-352) at 25°C with 12/12 h photo/dark period. Next, the culture was transferred to a 250 ml flask in an orbital shaker (Innova 44R) maintained at 120 rpm and 25°C with 12/12 h photo/dark period. After 7 days, 100 ml culture was inoculated into 900 ml growth media in 1-L cylindrical shape glass photobioreactors (PBR, 9 cm diameter), and it was illuminated from the side by white fluorescent lighting. The photosynthetic active radiation (PAR) value on the PBR glass wall was 500 μmol E/m^2^/s. A 1 ml pipette was used to pump air at the bottom of the PBR to have a mixing of 0.5 liter per minute. Three similar PBRs were used for this study and to produce adequate biomass for the subsequent experiments. The PBRs were kept in a temperature-controlled room maintaining 25 ± 2°C. After 10 days of cultivation, at the stationary phase, the biomass was harvested by centrifugation at 5,000 rpm for 5 min. The harvested biomass was then preserved in −80°C before extracting the pigment and preparation of the extract.

### Pigments Extraction and Quantification

The harvested biomass was freeze-dried with (Labconco FreeZone) freeze dryer. A known quantity of freeze-dried biomass sample and glass beads were added to a vial. Next, a known volume of 99.8% methanol HPLC grade was added to the vial. The vial was capped and placed in a bead beater for 15 min, followed by centrifugation at 5,000 rpm for 5 min. The pigments containing solvent decanted to another vial. If the biomass pellet color didn’t change extra methanol was added and the bead beating step repeated for maximum pigments extraction. Finally, the liquid was filtered through a PTFE syringe 0.2 μm for characterization. For the quantification of the pigments (Table 1), the absorbance of the supernatant obtained at 470, 652, and 665 nm using spectrophotometer DR3900 HACH Chlorophyll-*a* (Chl-*a*), Chlorophyll-*b* (Chl-*b*), and total carotenoids concentrations were calculated using equations (1), (2), and (3) respectively (21).


(1) $$Chla\left[\mu g.mL^{-1}\right]=\left[16.72\left(OD_{665}\right)-9.16\left(OD_{652}\right)\right]$$

(2) $$Chlb\left[\mu g.mL^{-1}\right]=\left[34.09\left(OD_{652}\right)-15.28\left(OD_{665}\right)\right]$$

$$Carotenoids\left[\mu g.mL^{-1}\right]$$

(3) $$=\frac{\left[1000\,\left(O\,D_{470}\right)-1.63\,\left(c\,h\,l\,a\right)-104.96\,\left(c\,h\,l-b\right)\right]}{221}$$

**TABLE 1:** Pigment quantification in the T1 crude extract.

Pigments	Carotenoids	Chl-*a*	Chl-*b*
Concentration (μg/ml)	527.20	243.55	138.41

The pigment was extracted from *H. pluvialis* biomass using methanol. The extracted pigment was then characterized using HPLC against the known standards of astaxanthin, lutein, and β-carotene (Figure 1B). The composition of pigment extracted from *H. pluvialis* biomass, in other studies (22), is similar to the composition obtained in this study. Other photopigments (e.g., chlorophyll, carotenoids) could also be present in the extract in minor concentrations. The pigment extract was concentrated under nitrogen flow by removing the bulk of the methanol. The concentrated pigment extract (T1) was used as it is for the experiment.

### Cell Culture

The aggressive BC cell line, MDA-MB-231 was obtained from the American Type Culture Collection (ATCC, Manassas, VA, United States), and the 3T3L normal fibroblast cells were provided by Weill Cornell Medicine-Qatar. Both MDA-MB-231 and 3T3L fibroblasts were cultured in DMEM supplemented with 10% fetal bovine serum (FBS), 1% penicillin, streptomycin, and 5% L-Glutamine. All the cell lines were cultured in the same optimum conditions in a humidified incubator adjusted to 37°C and 5% CO_2_. All the experiments were performed when cells attained 70–80% confluent.

### Alamar-Blue Cell Proliferation Assay

MDA-MB-231 and normal fibroblast 3T3L cell lines were seeded in 96-wells tissue culture plates with 10,000 cells in each well, in triplicates using DMEM medium supplemented with 10% fetal bovine serum (FBS) and 1% penicillin and streptomycin (100 μl/well).

Cells were incubated overnight prior to the treatment with at different concentrations of T1 methanolic extract (2.5, 5, 7.5, and 10%). Cells were also treated with methanol as a vehicle control for comparison. After 48 h, cells were washed with PBS and 10% Alamar-Blue reagent (Invitrogen, Thermo Fisher Scientific, Waltham, MA, United States), was added to the cells and incubated for 4 h in the incubator according to the manufacturer’s protocol. Fluorescence measurements were taken at 560 and 590 nm, using TECAN infinite M200 plate reader after incubation with the dye. Relative cell proliferation was determined based on the fluorescence of T1-treated cells relative to that of control cells.

### Morphological Study

MDA-MB-231 and 3T3L fibroblasts were treated with the optimal dose of 5% of T1 and were observed under the inverted microscope (OPTIKA Microscopes, Ponteranica, Italy) after 24 and 48 h of exposure to the treatment.

### Wound Healing Assay

MDA-MB-231 cells were seeded in 6-well plate and incubated overnight prior treatment. When the proper confluency was reached (∼50%), a straight scratch was made in each well using a sterile 200 μl tip. Cells were washed with sterile PBS to remove debris and unattached cells, and treated with 5% of T1 extract. Photos were taken at different time points during the treatment (0, 24 and 48 h) to monitor the width of the scratch. Images were analyzed using ImageJ software, statistical analysis, and the data were plotted as a graph.

### Invasion Assay

To determine the effect of T1 extract treatment of the invasive capacity of the BC cell, cell invasion assay was performed using the Boyden Matrigel Chamber assay. Cell invasion assay was carried out in 24-well Biocoat Matrigel invasion chambers (pore size of 8 μm, Corning, NY, United States) according to the manufacturer’s protocol. Briefly, untreated (control) and treated (5% of T1 extract as well as methanol) MDA-MB-231 cells were placed onto the upper chambers of Matrigel plates, and the bottom chamber was filled with DMEM medium containing 10% FBS as chemoattractant, and then incubated at 37°C. After 48 h incubation, the upper chambers were washed with sterile PBS and non-invasive cells were removed gently using a sterile cotton swab. Cells that invaded to the lower surface of the membrane were fixed with methanol and formaldehyde for 10 min and stained with 0.5% crystal violet. After washing out the stain with PBS, invaded cells were photographed under the inverted microscope (OPTIKA Microscopes, Ponteranica, Italy) in five predetermined fields. Percentage inhibition of invasive cells was calculated with respect to untreated cells and quantified using Image J software. Each experiment was carried out in triplicates.

### Western Blot Analysis

To identify the molecular pathways that are inhibited by T1 extract, the key molecular players associated with both cell cycle and apoptosis (p53, Bcl-2, and Bax) were investigated. Briefly, MDA-MB-231 cells were seeded and treated with 5% of T1 extract for 48 h. After 48 h of treatment, protein lysates were extracted using RIPA buffer and quantified by Bradford assay. Equal amounts (30 μg) of protein lysates were denatured at 95°C for 10 min and were resolved on 10% polyacrylamide gels and electroblotted onto PVDF membranes. The PVDF membranes were incubated overnight with the primary antibodies: anti-rabbit p53 (Cell Signaling Technology, CST #2527S, Danvers, MA, United States) anti-rabbit Bax (Cell Signaling Technology, CST #5023S, Danvers, MA, United States), anti-mouse Bcl-2 (Cell Signaling Technology, CST #15071S, Danvers, MA, United States). To confirm equal loading of protein samples, the membranes were re-probed with anti-rabbit β-actin (Cell Signaling Technology, CST #4970S, Danvers, MA, United States). Following overnight incubation, the membranes were washed twice in PBS and were then incubated for 2 h in the corresponding secondary antibody.

Immunoreactivity was detected by using ECL Western blotting substrate (Pierce Biotechnology, Rockford, IL, United States), as described by the manufacturer. Images were captured using the iBright machine (Thermo Fisher Scientific, Waltham, MA, United States) and relative quantification of protein expressions from images acquired from Western blotting were analyzed using ImageJ software. The intensity of the bands relative to the β-actin bands was used to calculate a relative expression of proteins.

### Statistical Analysis

The data were analyzed and graphs were plotted using GraphPad Prism software (version 8.4.3). Data were shown as an average of mean ± SEM (standard error of the mean). Each experiment was repeated at least three times (*n* = 3). One-way ANOVA followed by Tukey’s *post-hoc* test was used to compare the difference between treated and untreated cells. and differences with *p* < 0.05 were considered significant.

## Results

**FIGURE 2:**
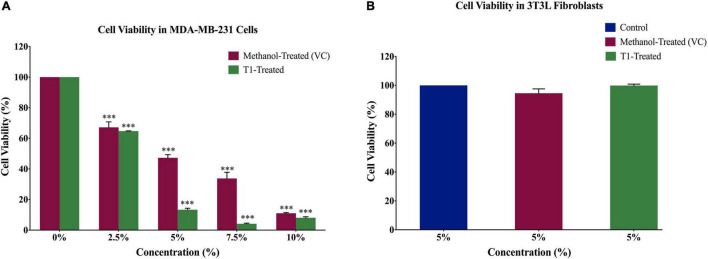
**(A)** Effect of different concentrations of T1 extract on cell viability of MDA-MB-231 cells after 48 h of treatment. Data indicate an inverse relationship between concentrations of T1-extract and cell proliferation in MDA-MB-231 cells as compared to the vehicle control (methanol). **(B)** Effect of T1 on cell viability of fibroblasts after 48 h of treatment. Data are presented as a percentage of treatment relative to the control (Mean ± SEM; *n* = 3). ***p < 0.0001. VC, Vehicle control.

In order to determine the optimal dose of T1 extract and subsequently optimize the experimental conditions for the BC cell line MDA-MB-231, as well as the control normal fibroblasts, cells were treated with varying concentrations (2.5, 5, 7.5, and 10%) of T1 extract for 48 h. In addition, 3T3L mouse fibroblast cells were used as control, while methanol was used as vehicle control (VC). Our results showed that T1 extract significantly reduced the number of proliferating MDA-MB-231 cells in a dose-dependent manner (Figure 2A) when compared to control cells; Notably, 5% of T1 extract showed a substantial decrease in MDA-MB-231 cell viability and was selected as the optimal dose for further investigation. Interestingly, the same optimal dose did not show any significant effect on the control cells (Figure 2B).

**FIGURE 3:**
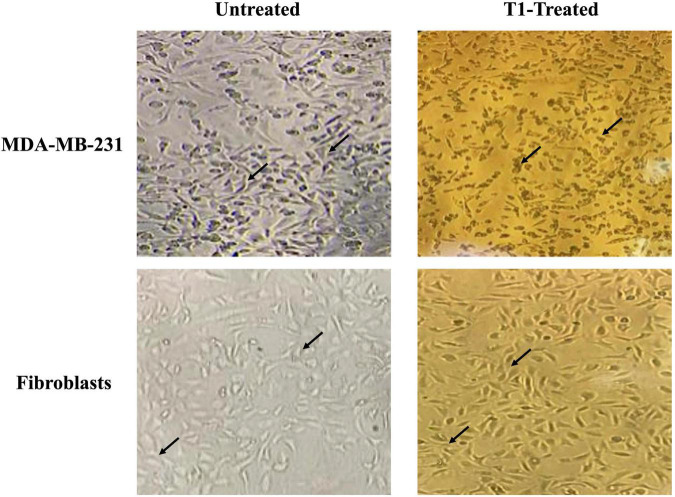
Effect of T1-extract on the morphology of MDA-MB-231. T1 induced cell death and the formation of a monolayer of cells in the MDA-MB-231 cells (black arrows indicate loss of cell-cell adhesion), in comparison with untreated (control) and fibroblast cells, which show no cytotoxic effect, displaying a round phenotype and form multilayers; Black arrows indicate epithelial morphology with clear cell-cell adhesion.

We further examined the morphology of MDA-MB-231 cells in addition to the control fibroblast cell lines under the effect of T1 extract treatment. In the absence of treatment, MDA-MB-231 cells displayed a smooth epithelial cell pattern with prominent nuclei. In comparison to their matched and vehicle controls, T1-treated MDA-MB-231 cell resulted in loss of cell-cell contact and detachment of cells from the surface of the tissue culture dish, indicating cell death or apoptosis; In contrast, this phenotype was not observed in the fibroblast control cells (3T3L), as shown in Figure 3.

**FIGURE 4:**
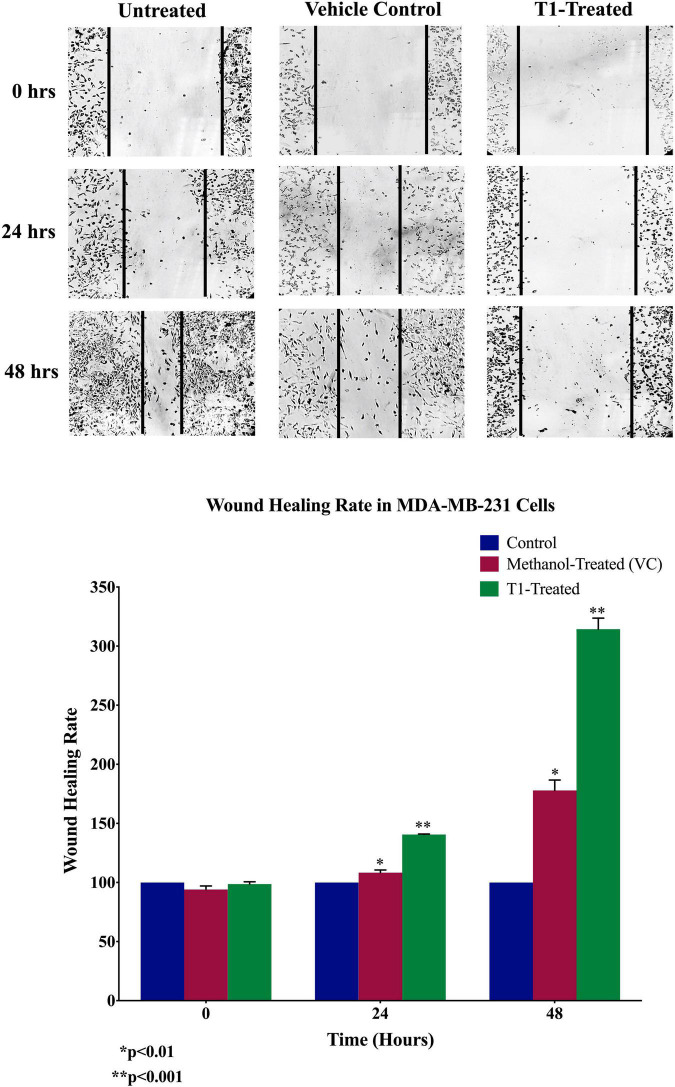
Effect of T1 on migration of MDA-MB-231 using wound healing assay. T1-extract sup-pressed cell motility of MDA-MB-231 cells in comparison to methanol treated (VC: vehicle control) and non-treated cells. Representative images are shown from three independent experiments and dark lines define the areas lacking cells (wound area, ImageJ). Values of percentage wound closure ± SEM (*n* = 3). *p < 0.01, **p < 0.001.

**FIGURE 5:**
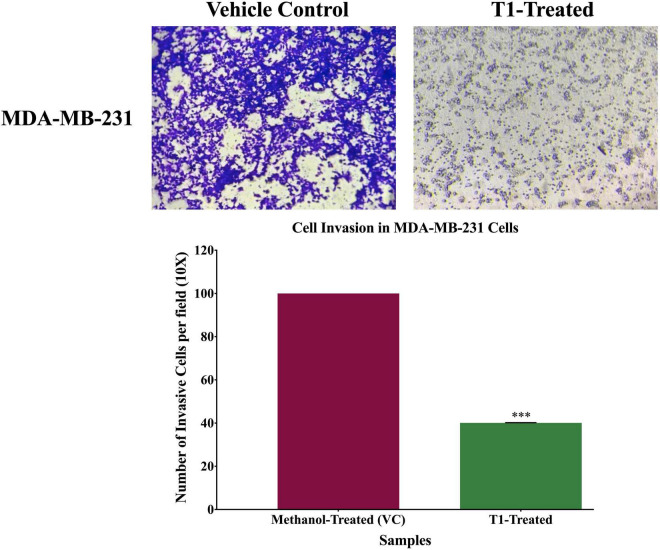
Effect of T1-extract on cell invasion of MDA-MB-231 breast cancer cell line using Boyden chamber assay. T1-extract significantly decreased cell invasion ability of MDA-MB-231 by ∼60% in comparison to control cells (VC: vehicle control) (***p < 0.0001).

Next, we analyzed the anti-migratory and anti-invasive effects of T1 extract on BC cells using wound-healing and invasion Boyden chamber assays, respectively. Our data revealed that T1 extract significantly inhibited cell migration and invasion of BC cells by ∼68 and 61%, respectively, in comparison to the control 48 h post-treatment (Figures 4, 5, *p* < 0.05).

**FIGURE 6:**
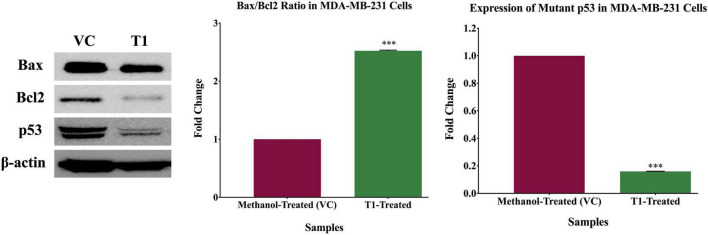
Expression patterns of proteins associated with T1-induced apoptosis. Western blot analysis of T1 extract- inhibited mutant p53 expression, while upregulating Bax/Bcl ratio, in comparison with their vehicle control (VC). β-actin was used as a control for the proteins amount in this assay. Cells were treated with 5% of T1-extract for 48 h, as explained in the materials and methods and the results sections. ***p < 0.0001.

Based on the observation that T1 extract induced cell death, we further explored the molecular mechanisms underlying this effect. Therefore, using western blot analysis, we investigated the expression patterns of the main apoptotic genes in T1 extract-treated cells, in comparison with their matched control (untreated) cells. Interestingly, the only band observed in the T1-treated fibroblasts control cells indicates the wild type p53, while the band observed in the T1- treated MDA-231 indicates both the wild-type and the mutant p53. Therefore, T1-treatment significantly reduced the expression of the mutant p53 in MDA-MB-231 compared to the T1-treated normal fibroblasts control cells (It is well-known that the MDA-231 cells express high levels of the stable mutant p53). Furthermore, T1 extract significantly increased the Bax/Bcl2 ratio (Figure 6).

Taken together, these data support our hypothesis that T1 extract plausibly inhibited cell growth and induced apoptosis, at least partially via loss of mutant p53 and increased Bax/Bcl2 ratio; This suggests that T1 extract induced apoptosis in BC cells *via* the mitochondrial p53/Bax/Bcl2 signaling pathway.

## Discussion

In the present study, we investigated the effect of T1 extract from *H. pulvialis* microalgae on proliferation, morphological changes, migration and invasion of the triple-negative human BC cell line (MDA-MB-231), compared to the normal fibroblast control cells. Furthermore, we explored the role of the major molecular signaling pathway mediating mitochondrial apoptosis in T1-treated cells. Our findings showed that T1 inhibited cell migration/invasion and induced apoptosis in MDA-MB-231 as compared to the normal fibroblast control cells. More interestingly, T1-induced apoptosis, appeared to be mediated at least via the mitochondrial p53/Bax/Bcl2 signaling pathway.

Various environmental factors including pH, temperature, nitrogen and phosphorus concentration significantly influence *H. pulvialis* biomass production as well as the chlorophyll content (13). Chlorophyll a and b are significant pigments for monitoring biomass growth in chlorophyta microalgae, especially in *H. pluvialis* as both chlorophylls adsorb different wavelengths in UV-visible spectrum (23). Studies have reported that although open pond reactors are simple and cost-effective systems for improving microalgae cultivation, there is a high chance of water loss due to evaporation and low growth rate due to lack of sufficient light, CO_2_ mass-transfer, and contamination (22). Thus, closed photo-bioreactors (PBRs) provide consistent CO_2_ mass-transfer and light intensities distribution, providing a suitable mean for microalgae cultivation (24). In addition, PBRs allow the recycling of the culture medium, thus reducing water consumption (25). Concordant to these findings, we extracted our pigment using PBRs.

There is accumulating evidence in the literature demonstrating that algae-derived bioactives exert anticancer activities, *via* induction of apoptosis, and inhibition of tumor cell invasion and metastasis (26–28). Among these bio-actives, the carotenoids astaxanthin (ATX) and fucoxanthin (FX) present in microalgae (29), possess anticancer characteristics and can inhibit cellular growth and restore the expression of tumor suppressor genes (28, 30, 31). In particular, FX inhibited cell growth of several types of cancers, including breast, colorectal, bladder, hepatocellular, leukemia and lymphoma (32). In combination with chemotherapeutic drugs, such as taxanes and doxorubicin, lutein reduced growth of BC and sarcoma cells, respectively (33, 34).

It is important to notice that carotenoids, including β-carotene, lutein, and canthaxanthin (CTX) are abundant in *H. pulvialis* (Table 1) (29). In fact, pigment analysis demonstrated a very high concentration of carotenoids in the T1 extract preparations as shown in Table 1. β-Carotene demonstrated anti-proliferative capabilities and self-renewal capacity of colon cancer stem cells by epigenetic alterations and global DNA methylation (35). Likewise, the carotenoid, ATX, inhibits cell metastasis, angiogenesis and arrests cell cycle at the G0/G1 phase *via* epigenetic modifications and/or chromatin remodeling (36, 37). On the other hand, Atalay et al. (36) reported that in combination with carbendazim, ATX suppressed BC proliferation *via* MCF-7 cells cycle arrest at the G2/M phase. Similar to ATX, lutein and FX impeded cell cycle progression (38). On the other hand, FX blocked cell cycle at the G0/G1 phase along with reduced cyclin D levels (28). Moreover, FX suppressed matrix metalloproteinase levels and inhibited cancer cell metastasis, in addition to induction of DNA degradation and apoptosis (28). Chang et al. (39) reported anti-proliferative role of lutein in BC *via* activation of the NrF2/ARE pathway and silencing of the NF-κB signaling pathway. Our findings are in concordance with previous findings (28, 36–39), suggesting that the carotenoid pigment in T1 extract might explain, at least partially, the anti-invasive effect of T1 extract on MDA-MB-231 cells.

ATX and FX stimulate apoptosis by suppressing the expression of the anti-apoptotic proteins and enhancing the expression of the pro-apoptotic proteins (28, 37). Likewise, CTX induced apoptosis in melanoma and colon cancer cells (40). On the other hand, lutein suppressed BC cell growth *via* increasing the intracellular ROS levels and induced p53-mediated apoptosis through leading to loss of Bcl2 expression (33). Concordant to the study by Gong et al. (33), our findings showed that T1 reduced cell growth and induced apoptosis of the human TNBC cells most likely via the p53/Bcl2 mitochondrial pathway. In fact, T1 has reduced the expression levels of the mutant p53 as shown in Figure 6, and increased the Bax/Bcl2 ratio. The mutant p53 is abundant in the TNBC MDA-MB-231 cells and suppresses apoptosis *via* a dominant negative effect (41). Also, Loss of p53 function increases the Bax/Bcl2 ratio, making cells more susceptible to mitochondrial apoptosis (42). More interestingly, although p53 is a tumor suppressor gene (43), certain mutations in p53 induce “dominant negative” or “gain-of-function” mutations which promote cell survival and tumorigenesis (44–47). In the BC cell line, MDA-MB-231 (41), mutant p53 is highly expressed and provides survival signals essential for cell survival and apoptosis by suppressing the effects of the proapoptotic members of the p53 family (48, 49). Thus, these data put together support our hypothesis that our findings that T1 crude extract exerts anti-proliferative effect by reducing the expression of the mutant p53 in MDA-MB-231, subsequently leading to the induction of apoptosis. Although, there are variety of *H. pluvialis* extracted products, research faces challenges to develop and scale up industrial production due to lack of economic support and productivity capacities (22, 50). Future work will include the extraction and fractionation of T1 crude extract in order to identify the major bioactive compound responsible for the anti-cancer activity in T1 for therapeutics.

## Conclusion

Despite advances in conventional diagnostic and therapeutics for cancer management, BC treatment remains challenging, particularly in triple-negative tumors. Our study showed the effect of *Haematococcus pluvialis* T1 crude extract on proliferation and invasion of the BC MDA-MB-231 BC cells, along with the molecular mechanisms associated with apoptosis. Our data revealed that T1 inhibition of the mutant p53 expression could be responsible for increasing the Bax/Bcl2 ratio leading to the induction of apoptosis. Thus, T1 might act as a candidate therapeutic agent based on its anticancer properties. Therefore, ongoing both *in vitro* and *in vivo* studies aim to validate the efficacy of T1-derived bioactives in suppressing BC tumor cell growth and progression.

## Data Availability Statement

The original contributions presented in the study are included in the article/supplementary material, further inquiries can be directed to the corresponding author.

## Author Contributions

AO: conceptualization, supervision, and funding acquisition. NA, SA, AF, MT, and PD: methodology. NA and AF: validation. NA: formal analysis and data curation. SA, NA, and IG: writing—original draft preparation. IG, A-EA, and AO: writing—review and editing. All authors have read and agreed to the published version of the manuscript.

## Conflict of Interest

The authors declare that the research was conducted in the absence of any commercial or financial relationships that could be construed as a potential conflict of interest.

## Publisher’s Note

All claims expressed in this article are solely those of the authors and do not necessarily represent those of their affiliated organizations, or those of the publisher, the editors and the reviewers. Any product that may be evaluated in this article, or claim that may be made by its manufacturer, is not guaranteed or endorsed by the publisher.
